# Turning Restriction Into Change: Imagine-Self Perspective Taking Fosters Advocacy of a Mandatory Proenvironmental Initiative

**DOI:** 10.3389/fpsyg.2019.02657

**Published:** 2019-11-29

**Authors:** Isabella Uhl-Haedicke, Johannes Klackl, Christina Muehlberger, Eva Jonas

**Affiliations:** ^1^Environmental Psychology, Department of Psychology, University of Salzburg, Salzburg, Austria; ^2^Social Psychology, Department of Psychology, University of Salzburg, Salzburg, Austria

**Keywords:** perspective taking, imagine-self, restriction, climate change, environmental attitude, environmental policy

## Abstract

Mandatory policies are needed to mitigate environmental problems but often elicit resistance if individuals perceive them as freedom restrictions. Encouraging people to take the perspective of individuals who suffer from environmental problems may help increase support. This should especially be the case with imagine-self as opposed to imagine-other perspective taking, because the former elicits more personal involvement than the latter. To test this hypothesis, we conducted two studies in which we announced the introduction of a voluntary vs. a mandatory proenvironmental initiative and asked people to take an imagine-self vs. imagine-other perspective on an individual, who suffers from human-caused environmental problems. The imagine-self condition increased the support of mandatory compared to voluntary initiatives. In addition, we found an influence of environmental attitude: the mandatory initiatives received higher support than voluntary initiatives by environmentally minded individuals. These findings highlight imagine-self perspective taking as a potentially useful tool for implementing proenvironmental policies.

## Introduction

“We have to wake up to the fierce urgency of the now,” said Jim Yong Kim, then president of the World Bank, stressing the necessity of acting on climate change, one of the biggest global problems of our time (Santiago, 2015). To avert the negative consequences of climate change, human societies need to become ecologically sustainable (Intergovernmental Panel on Climate Change, 2013). One approach is to force people to become eco-friendly by law. History shows that this strategy might be successful. For example, the Montreal Protocol in 1987 resulted in an international ban on chlorofluorocarbon gases because of their destructive effects on the ozone layer, which successfully decreased the atmospheric concentrations of these gases (United Nations Environmental Programme, 2016). Another example is catalytic converters, which became mandatory in many countries in the 1970s and 1980s to reduce automobile emissions of toxic gases, decreasing, for instance, carbon monoxide and nitrogen oxides. The public health benefits of catalytic converters far outweighed their costs (Hutchinson and Pearson, 2004; Palucka, 2004). Moreover, environmental taxes can also achieve the desired outcome. People in countries with high gasoline taxes tend to use a car less often than people in countries with low taxes (Von Weizaecker and Jesinghaus, 1992). The downside of these policies is that they can also easily backfire if individuals perceive them as restrictions of their personal freedom and rebel against them (Brehm, 1966; Brehm and Brehm, 1981). For example, planned road-pricing schemes aimed at reducing environmental problems and congestion have been rejected in Edinburgh and New York City due to the lack of public support (Gaunt et al., 2007; Schaller, 2010).

Optimizing public acceptance of environmental regulations is, therefore, a big challenge. Previous research suggests that promoting perspective taking with individuals involved in or affected by a restriction reduces resistance (Shen, 2010; Steindl and Jonas, 2012). In two studies, we tested to what extent imagine-self perspective taking (i.e., imagining oneself in another person’s situation) compared to imagine-other perspective taking (i.e., imagining another person in a specific situation) of victims of environmental problems would increase support of mandatory environmental actions that target these problems.

Imposing personal restrictions through environmental policies, regulations, and other kinds of governmental controls carries the risk of eliciting psychological reactance (Brehm, 1966; Brehm and Brehm, 1981). Reactance arises when individuals believe they possess a certain freedom but this freedom to decide and act in the moment or in the future is threatened. Reactance is a motivational state aimed at reestablishing freedom and regaining lost rights (Brehm, 1966; Brehm and Brehm, 1981). People reestablish their freedom either directly, by engaging in the opposite behavior of that demanded, or indirectly. Indirect freedom restoration can be achieved by observing others, who show the forbidden behavior or motivating others to engage in the behavior, or aggressively forcing the suppressor to remove the threat. Besides these behavioral reactions, reactance is accompanied by subjective reactions: restricted individuals experience anger or annoyance (Brehm, 1966; Brehm and Brehm, 1981; Dillard and Shen, 2005; Sittenthaler et al., 2015) and find the affected options more attractive than before (Brehm and Brehm, 1981; for an overview of reactance theory, see Miron and Brehm, 2006; Steindl et al., 2015; Muehlberger and Jonas, 2019). For example, recommendations to eat healthy food increased people’s choice of unhealthy alternatives (Fitzsimons and Lehmann, 2004), and learning about the health risks of smoking resulted in a greater desire for cigarettes and more positive attitudes toward smoking (e.g., Hylland and Birrell, 1979; Hansen et al., 2010). Unsurprisingly, reactance also arises in response to environmental freedom restrictions. For example, car users who experience a high level of freedom infringement have been shown to evaluate impending road pricing more negatively (Jakobsson et al., 2000), and the establishment of various conservation areas in Germany has induced strong opposition (Stoll-Kleemann, 2001; Ludwig et al., 2012).

People seem to be less resistant to absolute mandatory restrictions. An absolute mandatory restriction is certain and permanent, whereas a relative mandatory one is uncertain, temporary, and negotiable. In one study (Laurin et al., 2012), individuals who thought that the introduction of road pricing had a low implementation possibility (= relative mandatory) had more negative attitude toward the planned policy than individuals, who were made to believe that the probability was high (= absolute). The same pattern of results was found in other studies. Smoking bans in Switzerland gained acceptance over time in areas with strict regulations and were less accepted in areas with less stringent regulations (Rajkumar et al., 2014). Participants evaluated mobile phone use while driving more negatively when they thought that a ban on this behavior would definitely come into effect compared to those who did not (Laurin et al., 2012). Thus, it seems that when regaining a freedom seems impossible, reactance motivation and freedom-restoration efforts decrease (e.g., Brehm and Wright, 1983; Mikulincer, 1988). For this reason, absolute mandatory restrictions are associated with rationalization rather than reactance (Laurin et al., 2012; Proudfoot and Kay, 2014).

For governments intending to institute new environmental policies, the desired public response is rationalization rather than reactance. But policies are often discussed publicly before being passed into law, and climate policies change as they move through the legislative process. This elicits the impression that they are still negotiable and only temporary. Thus, the impression arises that public resistance might be able to successfully prevent or ban undesired bills. This breeds the chance of eliciting reactance.

Another factor that influences whether individuals respond with resistance to an environmental policy is environmental attitude. Environmentally friendly people typically demand and accept stronger environmental regulations such as fuel taxes or stronger building codes (Diekmann and Franzen, 1992) and sustainable transportation policies (Kim et al., 2013). We assume that environmentally aware people are more open to environmental restrictions, as an undamaged and clean ecosystem is important to them (Keuschnigg and Schubert, 2013). However, politicians depend on the voters’ positive evaluation if they plan to seek reelection, making it necessary to ensure acceptance among the whole population—including less environmentally friendly people.

One obstacle to implement new environmental policies is that humans are often unable to perceive slow environmental changes and consequently do not perceive the urgency of environmental agendas (Preuss, 1991). When people become aware of drastic environmental changes, their awareness of needing to act appears to rise. For example, the direct experience of climate change consequences through floods or air pollution can have a major influence on individuals’ proenvironmental actions (Whitmarsh, 2008). This indicates that the support of mandatory environmental policies among the general population should eventually increase as environmental problems become worse. At present, most Western societies are still not existentially affected by global environmental problems on a daily basis. Taking climate change as an example, most Western countries are midlatitude countries and have not yet experienced climate change consequences on a daily basis. Consequently, the motivation to act might fade in the face of other, seemingly more pressing problems in people’s daily lives. For instance, people might not perceive lower speed limits and higher fuel taxes as necessary and are thus more likely to experience reactance and show resistance toward these policies (Whitmarsh, 2008). Furthermore, they might vent their anger by voting out the politicians in charge. The politicians, in turn, may hesitate then to make environmental actions mandatory.

In sum, reactance is the enemy of mandatory environmental policies, and their perceived necessity is often low among people who have a low environmental attitude or are not directly affected by environmental problems. This might change if people experience the negative consequences of environmental pollution and/or climate change in their daily life. A potential technique to overcome this barrier could be perspective taking. Taking the perspective of individuals in regions that are already severely affected by human-caused environmental problems like climate change or pollution might result in a higher perceived necessity of environmental policies. Previous work suggests that perspective taking is indeed a promising political tool as it reduces reactance (Shen, 2010; Steindl and Jonas, 2012).

Perspective taking is defined as considering the world through the eyes of another, that is, from another’s viewpoint (Galinsky et al., 2005). It fosters positive relationships through more cooperative behavior (Parker and Axtell, 2001), inhibits interpersonal aggression (Richardson et al., 1994), facilitates forgiveness of offenses (McCullough et al., 1998), and reduces prejudices toward stigmatized groups (Galinsky and Moskowitz, 2001; Vescio et al., 2003). Previous research indicates that it can also function as an intervention to reduce reactance. In a study by Shen (2010), participants read persuasive messages that were aimed at reducing smoking or driving under the influence. Half of the messages contained empathy-inducing content (e.g., portraying victims suffering from lung cancer or car accidents) and the other half did not. Participants reported less reactance toward the persuasive messages when the messages induced state empathy with potential victims. Furthermore, taking the perspective of the person who imposed a freedom threat, and thus thinking about possible reasons for the threat, resulted in lower reactance (Steindl and Jonas, 2012). For this reason, perspective taking might also be a promising strategy to foster public support of environmental regulations.

There are two ways of perceiving another’s perspective (Batson et al., 1997): People can imagine either how the other person experiences a situation (imagine-other) or how they would experience the situation themselves if they were in the other person’s position (imagine-self). These two perspectives elicit distinct reactions that might be useful in environmental politics because they have different consequences (Stotland, 1969; Batson et al., 1997; Batson, 2014). In an experiment by Batson et al. (1997), individuals read a story about a university student, Katie Banks, who struggled to take care of her brother after their parents passed away. Half of the participants imagined how Katie was feeling in the situation (imagine-other) and the others imagined how they themselves would feel if they were in Katie’s position (imagine-self). People in both conditions expressed a high level of empathy, which results from the awareness of another person’s suffering and a sense of responsibility to alleviate this suffering (Stern et al., 1993). However, only people in the imagine-self condition reported a feeling of personal distress, which is thought to awaken the egoistic motivation to relieve one’s own distress (Batson, 2014).

Feelings of empathy that arise during both imagine-other and imagine-self perspective taking usually stimulate the altruistic motivation to improve the situation of the person for whom empathy is felt (Stotland, 1969; Batson et al., 1997; Batson, 2014). By vicariously experiencing other individuals’ distress, people may acknowledge the urgency of environmental regulations, and hence, increase their support of proenvironmental policies. Previous research indeed confirms that empathy for animals affected by environmental problems (e.g., a seal caught in a fishing net, a dead bird on the beach covered in oil) arouses strong environmental attitudes and behavior (Schultz, 2000; Berenguer, 2007).

Imagine-self perspective taking may be the key to elicit distress among the entire population rather than just among the people who are most severely affected (Batson, 2014). An egoistic motivation to resolve negative environmental consequences for oneself should stimulate concern for environmental problems (Schultz, 2000). For this reason, we predict that following mandatory environmental regulations, people exposed to imagine-self perspective taking will exhibit higher support than people exposed to imagine-other perspective taking, because of the additional desire to reduce personal distress by acting proenvironmentally. In the current studies, we tested this assumption.

The aim of the current research was to test the effects of the two types of perspective taking (imagine-other vs. imagine-self) as well as proenvironmental attitudes on support of environmental regulations. We told participants in Studies 1 and 2 about a new initiative aimed at making their university more sustainable and appealing. They either had to participate in the initiative (mandatory conditions, which were either an absolute mandatory or a relative mandatory restriction in Study 1 and a relative mandatory restriction in Study 2) or were allowed to participate voluntarily (voluntary condition). In all conditions, the initiative included eco-friendly actions such as organizing a vegetarian event that highlighted the negative impact of meat consumption on the environment, making sure that the lights and projectors were turned off in the evening in the seminar rooms, or collecting and recycling garbage around campus. Then, they watched a short movie focusing on different environmental problems and were told to take the perspective of an individual (imagine-other vs. imagine-self) who appeared in the film. The characters in the films were threatened by contamination of the oceans with plastics (Study 1) or by rising sea levels caused by climate change (Study 2); sustainable, proenvironmental actions would mitigate their problems. The pollution of the oceans and climate change constitute two of the biggest environmental problems. We chose these topics as the findings might be of high practical value. Finally, we assessed participants’ evaluation of the initiative’s actions.

1. Furthermore, we tested the influence of two types of perspective taking. We predicted imagine-self perspective taking would have a stronger effect (i.e., lead to higher support for the initiative) than imagine-other perspective taking following a mandatory restriction (vs. a voluntary restriction) because of the additional experience of personal involvement (cf. Batson et al., 1997). Imagining oneself in the position of a person already suffering from human-caused environmental problems should increase the motivation to solve the problem by showing a higher degree of support for the actions in the mandatory condition than in the voluntary condition (*perspective-taking hypothesis*).
2. In addition, we investigated the role of proenvironmental attitudes following a mandatory restriction. Environmentally friendly people have been shown to respond with a higher support for environmental regulation (Diekmann and Franzen, 1992; Kim et al., 2013). Furthermore, previous research indicated that confronting environmentally friendly people with environmental problems, such as the videos presented in Studies 1 and 2, seemed to work as a boost. It resulted in a higher willingness to act in an environmentally friendly way compared to environmentally friendly people who did not get the environmental message (Bolderdijk et al., 2013; Uhl et al., 2016). Therefore, and in line with previous research (Diekmann and Franzen, 1992; Bolderdijk et al., 2013; Kim et al., 2013; Uhl et al., 2016), we expected highly proenvironmental people to evaluate the actions even more positively in the mandatory condition than in the voluntary condition (*environmental attitude hypothesis*).

## Study 1

### Method

#### Participants and Design

Participants were approached on campus or *via* the university mailing list and invited to voluntarily participate. We recruited 110 students from the University of Salzburg. One participant was excluded from the sample because of missing data. This resulted in a final sample of 109 participants (78 women, 30 men, 1 unidentified; *M*_age_ = 23.75 years, SD = 8.30). Participants were randomly assigned to one of the three freedom restriction conditions (absolute mandatory vs. relative mandatory vs. voluntary) and a perspective-taking condition (imagine-other vs. imagine-self). This resulted in a 3 (freedom restriction: absolute mandatory vs. relative mandatory vs. voluntary) × 2 (perspective taking: imagine-other vs. imagine-self) between-subjects experimental design.

#### Measures and Procedure

**Figure 1:**
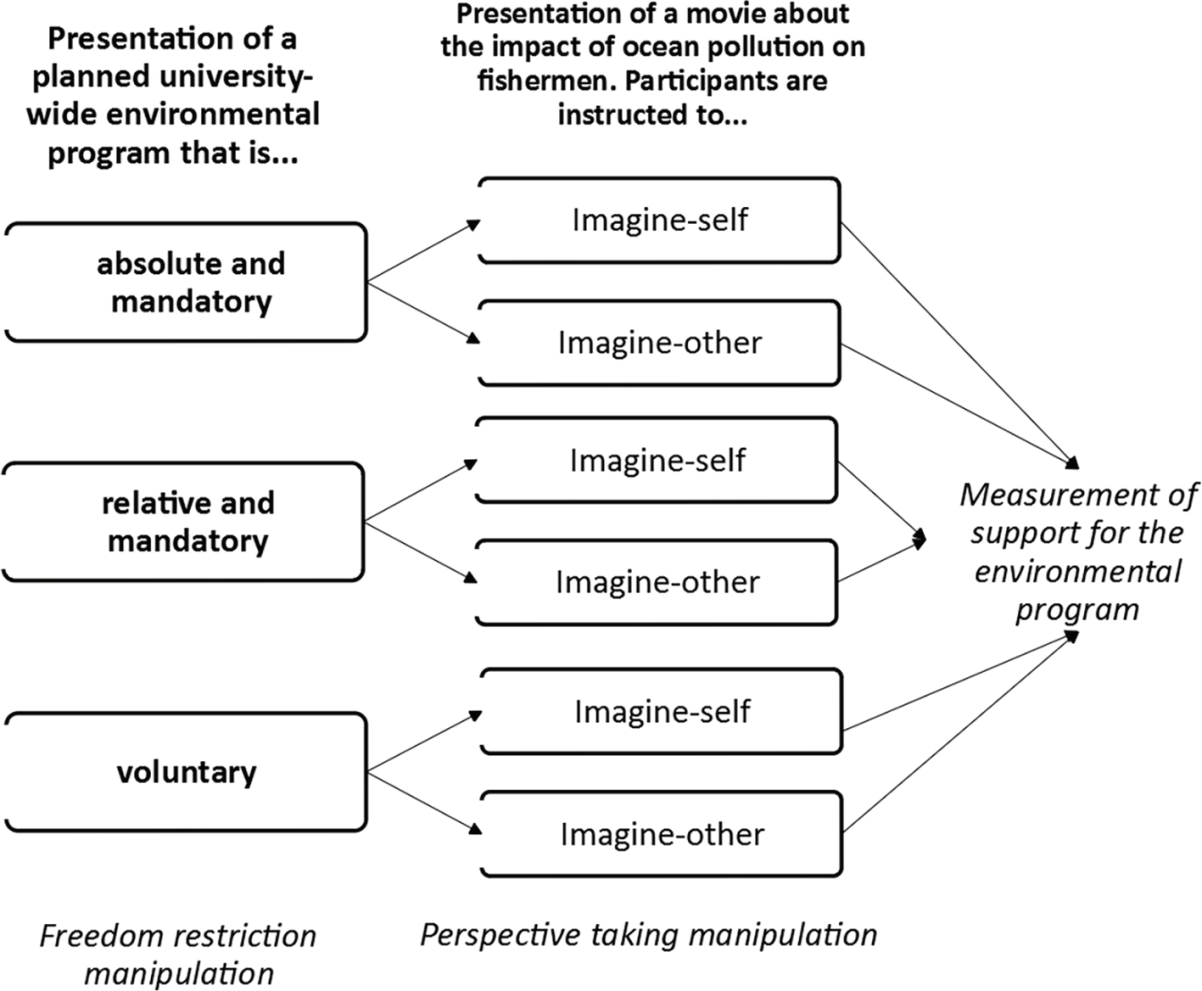
Design of study 1.

As a cover story, we told the participants that we were interested in their evaluation of a new initiative and a movie. Participants first provided informed consent and demographic information and then read about an initiative aimed at making the university more sustainable and appealing (the freedom restriction manipulation). After this, participants watched a movie about ocean pollution and imagined that they or someone else was affected by it (perspective-taking manipulation). Finally, they evaluated the actions of the initiative and were fully debriefed, thanked, and dismissed (see Figure 1). We also administered a personal distress scale (Batson et al., 1997) and 10 items of the Positive and Negative Affect Schedule (PANAS; Watson et al., 1988) and assessed ease of the perspective-taking task. The self-reported measures are not relevant for the present manuscript but results can be requested from the authors.

#### Freedom Restriction Manipulation (Voluntary vs. Mandatory Actions)

The aim of the initiative was to improve the university through various actions, most aimed at promoting ecological sustainability (e.g., collecting plastic bottles at the university, collecting garbage outside the university). In the voluntary restriction condition, students (*n* = 33) were made to believe that they could participate voluntarily in the actions. There were two mandatory conditions: In the absolute mandatory restriction condition, students (*n* = 33) were made to believe that they were obligated to participate in the actions; in the relative mandatory restriction condition, students (*n* = 43) were made to believe that they were obligated to participate in the actions during a testing period of 6 months, after which the initiative would be reevaluated.

#### Reactance Measure

Building on the classic measure of reactance, that is, the decrease in attractiveness of an imposed option (Brehm, 1966; Brehm and Brehm, 1981), we assessed participants’ ratings of the initiative’s actions. To provide another measure of reactance, we used items of the validated Salzburger State Reactance Scale (SSR Scale; Sittenthaler et al., 2015). On a scale ranging from 1 (*not at all*) to 5 (*very much/strong*), participants evaluated their current experience of reactance (*α* = 0.90, seven items, *M* = 2.33, SD = 0.69, e.g., “To what extent do you perceive the implementation of the initiative as a restriction of freedom?”) and behavioral intentions (*α* = 0.80, four items, *M* = 1.88, SD = 0.92, e.g., “How strong is your desire to complain about the implementation to the curricular committee?”).

#### Imagine-Self vs. Imagine-Other

For our between-subjects manipulation of perspective taking, we showed participants a short movie (*Garbage Islands*) about worldwide pollution of the oceans (Victor et al., 2013). We told them to follow the movie carefully because they would have to answer questions about it later on. Before watching the movie, they were instructed either to imagine the feelings and thoughts of a fisherman who was affected by the pollution in the region mentioned in the movie (imagine-other condition, *n* = 55) or to imagine themselves being fishermen in the region (imagine-self condition, *n* = 54). After watching the movie, participants wrote down their thoughts and feelings. To keep up the cover story, we asked 11 multiple choice and open format questions about the movie the participants had watched.

#### Evaluation of Actions

Next, participants were asked to evaluate the planned initiative. Participants were presented with a list of 15 actions (see Table 1), most aimed at promoting ecological sustainability. They rated on a scale of 1 (*not at all*) to 5 (*very much*), their willingness to perform the actions (*M* = 2.81, SD = 0.77; *α* = 0.87) and how important they thought the actions were (*M* = 3.40, SD = 0.68; *α* = 0.86). The two measures were highly correlated [*r*(106) = 0.60] and thus combined into a single measure of attractiveness (*M* = 3.10, SD = 0.65; *α* = 0.91).

**Table 1:** Items used in Study 1 to evaluate the planned University initiative.

Collecting garbage inside the University building
Collecting garbage outside the University building
Cleaning the University’s pond
Winter service (laying anti-slip mats spreading salt in the morning)
Cleaning windows (twice per semester) and attach bird silhouettes
Collect and recycle plastic bottles in the University building
Remove graffitis from toilet walls
Table service in the cafeteria: cleaning the tables
Composting service (Setting up a compost heap and turning it to distribute microorganisms)
Water plants and repot them in spring
Decorating the walls through an art project
Removing chewing gum from under the seats in the lecture halls
Turning off the heaters in one floor of the University building in the evening
Turning off the lights in one floor of the University building after 6 pm
Arrange holiday-themed decoration (e.g., Christmas stars)

#### Proenvironmental Attitude

To assess participants’ environmental attitude, we presented eight statements regarding the severity of environmental problems (Keuschnigg and Schubert, 2013). They rated on a scale of 1 (*not at all*) to 5 (*very much*) to what extent they agreed with each statement (*M* = 3.74, SD = 0.60; *α* = 0.63, e.g., “I am worried when I think about the future environment my children and grandchildren potentially have to live in”).

#### Data Analysis

To assess the effects of the experimental manipulations and individual environmental behavior on the outcomes of interest, we used a linear regression analysis. We coded the mandatory freedom restriction manipulations using two dummy-coded variables, one for the absolute mandatory (=REA) and one for the relative mandatory restriction (=RER) condition. The voluntary condition was used as the reference category. The perspective-taking manipulation was coded such that 1 stood for the imagine-self condition and 0 for the imagine-other condition. Proenvironmental attitude was centered prior to entering it as a predictor. Interactions were followed up with simple slopes analyses, using the pequod package in R (Mirisola and Seta, 2016).

### Results

#### Reactance

First, we tested whether and to what extent the freedom restriction manipulations evoked feelings of reactance, as indicated by the SSR Scale. Although neither the relative mandatory nor the absolute mandatory freedom restriction condition led to significantly elevated reactance feelings, the results were in the predicted direction, RER: *b* = 0.15, SE = 0.23, *t*(107) = 0.67, *p* = 0.50; REA: *b* = 0.38, SE = 0.21, *t*(107) = 1.74, *p* = 0.08; namely, participants in the mandatory freedom restriction conditions experienced more reactance (REA: *M* = 2.28, SD = 0.94, RER: *M* = 2.51, SD = 0.93) than those in the voluntary freedom restriction condition (*M* = 2.13, SD = 1.00). Looking at the behavioral intention subscale, there was no significant difference between participants in the mandatory freedom restriction conditions (REA: *M* = 1.96, SD = 0.80, RER: *M* = 1.89, SD = 0.90) and those in the voluntary freedom restriction condition, *M* = 1.74, SD = 1.09; RER: *b* = 0.23, SE = 0.21, *t*(107) = 1.10, *p* = 0.30; REA: *b* = 0.16, SE = 0.23, *t*(107) = 0.69, *p* = 0.50. These results suggest that the mandatory versions of the initiative did not provoke significantly stronger feelings of reactance nor higher intentions to take action against the initiative than the voluntary version.

#### Main Analysis

Next, we tested our main hypothesis that imagine-self participants in the mandatory freedom restriction conditions would evaluate the actions more positively than imagine-self participants in the voluntary condition. Furthermore, we examined the environmental attitude hypothesis that highly environmentally friendly individuals in the mandatory freedom restriction conditions would evaluate the actions more positively than highly environmentally friendly individuals in the voluntary condition. The results of the regression analysis are displayed in Table 2. We did not find any main effect of mandatory relative mandatory or absolute mandatory freedom restriction, perspective taking, or environmental attitude.

**Table 2:** Moderation analyses for variables predicting action evaluation (*N* = 109) in Study 1.

Variable	*b*	SE	*t*(100)	*p*
Intercept	2.90	0.13	22.16	0.00
Absolute freedom restriction	0.18	0.19	0.94	0.35
Relative freedom restriction	−0.07	0.18	−0.39	0.70
Perspective taking	−0.09	0.19	−0.46	0.65
Environmental attitude	−0.07	0.15	−0.50	0.62
**Absolute restriction × Environmental attitude**	**0.72**	**0.21**	**3.50**	**<0.001**
**Relative** mandatory **restriction × Environmental attitude**	**0.75**	**0.23**	**3.27**	**<0.001**
Absolute mandatory restriction × Perspective taking	0.18	0.27	0.65	0.52
**Relative** mandatory **restriction × Perspective taking**	**0.66**	**0.25**	**2.58**	**0.01**

*Significant predictors are indicated in bold*.

**Figure 2:**
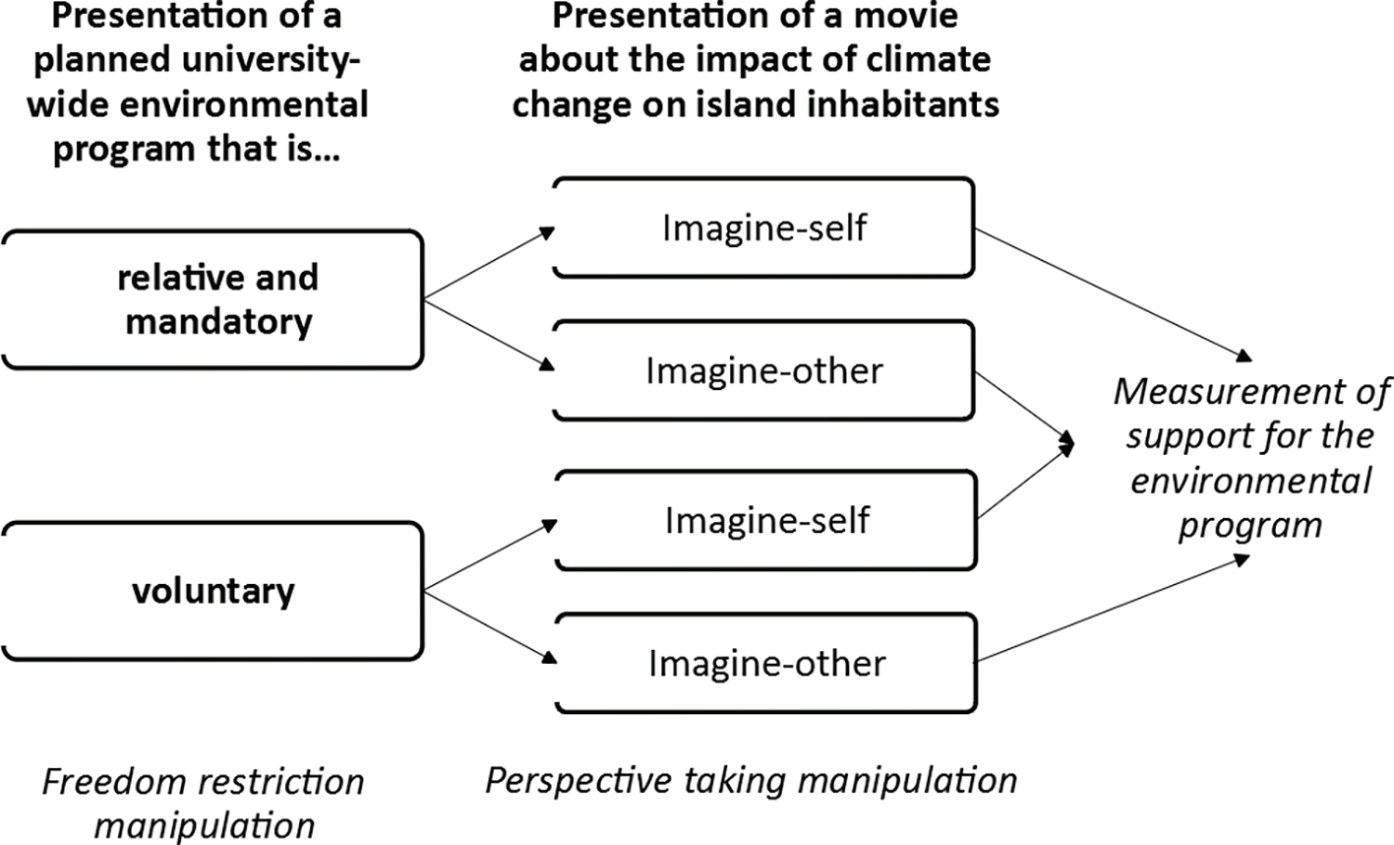
Design of study 2.

Most importantly for our hypothesis, we found an interaction between relative freedom restriction and perspective taking. The relative mandatory freedom restriction increased the attractiveness of the program, especially in the imagine-self condition, *b* = 0.58, SE = 0.18, *t*(100) = 3.16, *p* = 0.002 (see Figure 2). The effect in the imagine-other condition was not significant, *b* = −0.06, SE = 0.17, *t*(100) = −0.39, *p* = 0.69 (see Figure 2). Note that this pattern of effects was specific to the relative mandatory restriction condition and did not emerge with the absolute mandatory restriction, imagine-other: *b* = 0.17, SE = 0.18, *t*(100) = 0.94, *p* = 0.35; imagine-self: *b* = 0.35, SE = 0.19, *t*(100) = 1.83, *p* = 0.07. This indicates that imagine-self perspective taking benefits the support for a mandatory program when the program is framed in a relative mandatory fashion.

Furthermore, the interactions between absolute freedom restriction and environmental attitude and between relative freedom restriction and environmental attitude were significant (Table 2). For high environmentally friendly people (+1 SD), both restrictions increased evaluations of the initiative’s actions, relative: *b* = 0.78, SE = 0.30, *t*(100) = 2.60, *p* = 0.01; absolute: *b* = 0.99, SE = 0.29, *t*(100) = 3.38, *p* = 0.001, relative to the voluntary condition. For less environmentally friendly individuals, the reverse was true, relative: *b* = −1.47, SE = 0.47, *t*(100) = −3.09, *p* = 0.002; absolute: *b* = −1.16, SE = 0.43, *t*(100) = −2.68, *p* = 0.008.

#### Discussion

The aim of the study was to test the influence of perspective taking (imagine-other vs. imagine-self) and environmental attitude on the response to an environmental initiative. In line with our prediction, we found that participants in the relative mandatory condition tended to evaluate the environmental initiative more positively if they had earlier put themselves in the shoes of a fisherman whose life was affected by the environmental problems that the initiative was trying to address. This finding is in line with previous research that identified perspective taking as an intervention to minimize resistance to a freedom threat (Shen, 2010; Steindl and Jonas, 2012). Furthermore, environmentally friendly individuals gave higher evaluations of the actions of the initiative if they had to participate (i.e., in the absolute mandatory and relative mandatory conditions) compared to participating on a voluntary basis. Less environmentally friendly participants exhibited the opposite pattern.

However, since framing the initiative as mandatory did not elicit reactance in the first place (except for less environmentally friendly individuals), we cannot conclude that perspective taking actually turned resistance into support; rather, it merely magnified the support that was already present. Therefore, in Study 2, we used a stronger freedom restriction manipulation. Again, we hypothesized that imagine-self individuals in a mandatory condition would evaluate the actions more positively than imagine-self individuals in a voluntary condition. Furthermore, we examined the environmental attitude hypothesis that highly environmentally friendly individuals would evaluate the actions more positively in the mandatory condition than in the voluntary condition.

## Study 2

Since we found the interaction with perspective taking in the relative mandatory restriction condition only, we omitted the absolute mandatory freedom restriction condition of Study 1. We also deemed it not fully representative of the political process. In reality, (environmental) policies might change after public discussion, during the legislative process, following demonstrations, or due to new administrations.

Furthermore, we used a different movie that points out the devastating impact of climate change to rule out that the effects were limited to the particular topic or movie employed in Study 1. Furthermore, we replaced some actions of the initiative with actions that matched the topic of the new video (e.g., Study 1: “Cleaning the pond,” Study 2: “Organizing a vegetarian festival to raise awareness for the high CO_2_ emissions caused by meat production”; Study 1: “Collecting plastic bottles at the university,” Study 2: “Organizing an event about climate change”).

### Method

#### Participants and Design

A 2 (restriction of freedom: voluntary vs. mandatory) × 2 (perspective taking: imagine-self vs. imagine-other) between-subjects experimental design was employed. Individuals were approached on campus or *via* the university mailing list and asked to participate voluntarily. We recruited 65 students (55 women, 10 men; *M*_age_ = 23.85 years, SD = 9.22).

#### Measures and Procedure

**Figure 3:**
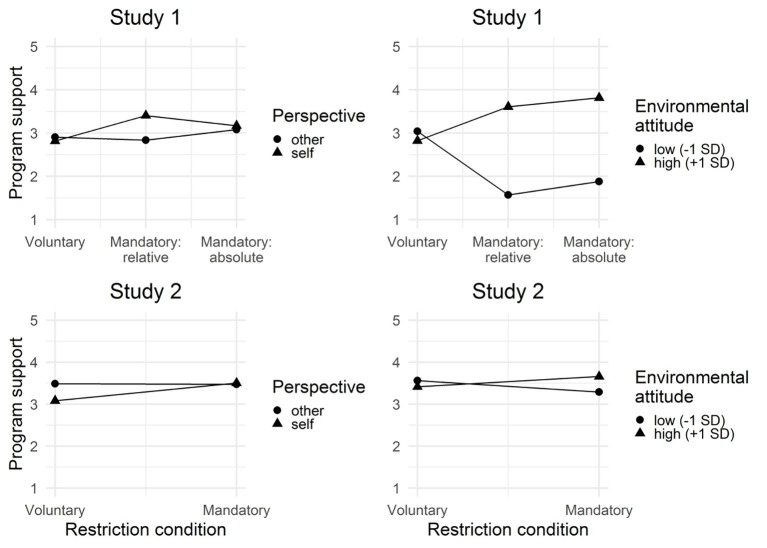
Mean evaluations of the environmental program initiative by freedom restriction condition (Study 1: voluntary, mandatory: relative, mandatory: absolute; Study 2: voluntary, mandatory) and perspective-taking condition (imagine-other, imagine-self) and by environmental attitude (low, high friendliness).

The procedure was similar to that of Study 1. Participants first were informed about the voluntary or mandatory initiative and watched a movie about the consequences of climate change during which the perspective-taking manipulation took place. Then, they evaluated the actions of the initiative (see Figure 3). Finally, they were fully debriefed, thanked, and dismissed. We also administered a personal distress scale (Batson et al., 1997) and the PANAS (Watson et al., 1988), assessed participants’ evaluation of human influence on climate change, and administered the Behavior Inhibition Scale and the Behavior Activation Scale (Carver and White, 1994) and the Self-Esteem Scale (Hagborg, 1993). The measures are not relevant for the present manuscript but results can be requested from the authors. We also collected electroencephalographic (EEG) data. Due to problems with the EEG recording device, the data were of low quality and could not be analyzed.

#### Voluntary vs. Mandatory Actions

After providing demographic data, participants read about the initiative. We randomly assigned participants to the voluntary (*n* = 32) or mandatory (*n* = 33) condition. As in Study 1, we afterward assessed participants’ experience of reactance and behavioral intentions (Sittenthaler et al., 2015; experience of reactance: *M* = 1.93, SD = 0.70; *α* = 0.85; behavioral intentions: *M* = 1.47, SD = 0.49; *α* = 0.71).

#### Imagine-Self vs. Imagine-Other

We next showed a movie featuring the inhabitants of the island nation Kiribati, who point out how they are affected by climate change. Kiribati will disappear due to the rising sea level (DW TV, 2009). Participants were instructed to imagine their feelings and thoughts if they themselves were inhabitants of the island (imagine-self, *n* = 32) or to imagine the feelings and thoughts of the inhabitants (imagine-other, *n* = 33) while watching the movie. After finishing the movie, participants wrote down their thoughts and feelings and took a quiz about the movie to keep up the cover story.

#### Action Evaluation

Next, participants evaluated a list of 18 actions (modified and extended from the list used in Study 1; see Table 3). To strengthen the freedom restriction manipulation, we reiterated the voluntary or mandatory nature of the initiative. The participants rated on a scale of 1 (*not at all*) to 5 (*very much*) their willingness to conduct each action (*M* = 3.17, SD = 0.56; *α* = 0.78) and how important they thought the action was (*M* = 3.43, SD = 0.49; *α* = 0.77), resulting in an action evaluation scale [*M* = 3.30, SD = 0.48; *α* = 0.86, *r*(63) = 0.60].

**Table 3:** Items used in Study 2 to evaluate the planned University initiative.

Organizing an event on climate change
Composting service (Setting up a compost heap and turning it to distribute microorganisms)
Arrange holiday-themed decoration (e.g., Christmas stars)
Water plants and repot them in spring
Help organize the University’s Christmas party
Collecting and recycling garbage inside the University building
Help organize a vegetarian event to raise awareness of the high CO_2_ emissions caused by meat production
Help organize a funding campaign to support students in need
Help set up a free tutoring platform for students
Help organize a blood donation event at the university
Appoint students who turn off the lights and video projectors in one floor of the University building in the evening
Help organize a funding campaign for the University project ‘Renewable Energy for Africa with the help of solar energy’
Supervising a new recreation area for students
Help set up and administer a University carpool platform
Help organize a charity Christmas market at the University
Collecting garbage collection outside the University building
Decorating the walls through an art project
Winter service (laying anti-slip mats spreading salt in the morning)

#### Proenvironmental Attitude

We used the same scale as in Study 1 to assess participants’ general environmental attitude (Keuschnigg and Schubert, 2013; three items, *M* = 3.93, SD = 0.58; *α* = 0.59).

#### Data Analysis

We ran a moderation analysis with freedom restriction (voluntary vs. mandatory; voluntary = 0, mandatory = 1) and perspective taking (imagine-other vs. imagine-self; other = 0, self = 1) as the independent variables, (mean-centered) proenvironmental attitude as a moderator, and the action evaluation as dependent variable.

### Results and Discussion

#### Reactance

First, we tested whether the freedom restriction manipulation evoked feelings of reactance. Participants in the mandatory condition (*M* = 2.21, SD = 0.80) experienced a significantly higher level of reactance compared to the participants in the voluntary condition (*M* = 1.64, SD = 0.44), *t*(63) = 3.56, *p* < 0.001. The analysis for behavioral intention also showed a significant difference between participants in the mandatory condition (*M* = 1.61, SD = 0.53) and the voluntary condition (*M* = 1.34, SD = 0.41), *t*(59) = 2.27, *p* = 0.02.

#### Main Analysis

Again, we tested our main hypothesis that imagine-self participants in the mandatory condition would evaluate the actions more positively than imagine-self participants in the voluntary condition. Furthermore, we examined the environmental attitude hypothesis that highly environmentally friendly individuals in the mandatory condition would evaluate the actions more positively than highly environmentally friendly individuals in the voluntary condition. The results of the regression analysis are displayed in Table 4.

**Table 4:** Moderation analyses or variables predicting action evaluation (*N* = 65) in Study 2.

Variable	*b*	SE	*t*(63)	*p*
Intercept	3.50	0.10	36.39	< 0.001
Freedom restriction	−0.05	0.14	−0.35	0.73
**Perspective taking**	**−0.41**	**0.14**	**−2.94**	**0.004**
Environmental attitude	−0.13	0.13	−1.00	0.32
**Freedom restriction × Perspective taking**	**0.43**	**0.19**	**2.27**	**0.03**
**Freedom restriction × Environmental attitude**	**0.44**	**0.17**	**2.63**	**0.01**

*Significant predictors are indicated in bold*.

We did find a main effect of perspective taking. People in imagine-other condition evaluated the initiative more positively than people in the imagine-self condition. We did not find a main effect of freedom restriction or environmental attitude.

In line with our predictions, the interaction between freedom restriction and perspective taking was significant (see Figure 2). Again, the mandatory freedom restriction increased the attractiveness of the program in the imagine-self condition, *b* = 0.42, SE = 0.13, *t*(59) = 3.10, *p* = 0.003, but not in the imagine-other condition, *b* = −0.01, SE = 0.13, *t*(59) = −0.10, *p* = 0.92. Interestingly, the imagine-self perspective taking reduced the attractiveness of the voluntary program more than it increased the attractiveness of the mandatory program.

Similar to in Study 1, high environmentally friendly participants in the mandatory condition evaluated the actions more positively than high environmentally friendly individuals in the voluntary condition, *b* = 0.24, SE = 0.15, *t*(59) = 1.59, *p* = 0.12, whereas the reverse was true for low environmentally friendly participants: They tended to evaluate the mandatory program as more negative, *b* = −0.27, SE = 0.17, *t*(59) = −1.54, *p* = 0.13. Neither effects were significant and they should only be interpreted with caution.

#### General Discussion

Our goal was to investigate the impact of two types of perspective taking (imagine-other vs. imagine-self) and environmental attitude on the support of mandatory environmental initiatives. We told participants in Studies 1 and 2 about voluntary vs. mandatory participation in a sustainability initiative. As a potential resistance-buffering intervention, they had to take the perspective (imagine-other vs. imagine-self) of an individual already severely affected by human-caused environmental problems. Finally, we assessed their evaluation of the initiatives’ actions.

In line with our prediction, we found that participants in both studies evaluated the mandatory tasks more positively if they imagined themselves in the position of a person already affected by climate change. We attribute this to the stronger personal involvement that imagine-self perspective taking is able to bring about. According to past research, both perspective-taking techniques should induce empathy and an altruistic motivation to reduce others’ distress, but imagine-self perspective taking should additionally induce personal distress, and an egoistic motivation to reduce that personal distress (Stotland, 1969; Batson et al., 1997; Batson, 2014). If supporting the environmental program would have been purely altruistically motivated, people should have supported the program to similar amounts in both perspective taking conditions, as both evoke empathy. However, our finding that imagine-self perspective taking was able to raise additional support for the environmental program indicates that personal distress and the resulting egoistic motive to reduce it is crucial.

However, it seems that to raise motivation to act on human-caused environmental problems such as pollution and climate change, self-involvement through the direct experience of environmental problems is needed (Whitmarsh, 2008). People are not able to perceive the ozone hole or greenhouse gases in the atmosphere. Even severe changes such as species extinction, melting of the polar ice, or suffering of island inhabitants often go unnoticed by Western society, as such changes are at the moment mainly happening in remote places. Very often, people perceive the consequences only when the human behavior has already caused severe damage. Waiting for people in the Western world to experience the severity of pollution and climate change on a daily basis does not sound like a promising strategy, and taking restorative action to minimize the consequences of climate change might already be too late. Our results indicate that imagine-self perspective taking might be a promising technique for getting people to accept environmental initiatives. As imagine-self perspective taking elicits personal distress, leading people to seek resolutions the situation (Stotland, 1969; Batson et al., 1997; Batson, 2014), it may help close the time gap between the execution of behavior harmful to the environment and its consequences. Participants in the imagine-self condition expressed higher support for mandatory (vs. voluntary) environmental actions in both of our studies. This was the case even though the consequences occurred in other parts of the world with no immediate influence on participants’ daily lives. We assume that this is what is needed in Western societies to promote support for environmental policies in the now.

As expected, high environmentally friendly participants responded in a more positive way if the actions were mandatory. In both studies, they evaluated the actions of the initiative more highly when they were made to believe that they were being forced to participate compared to when they were made to believe that they could choose to participate. These results are in line with previous research (Bolderdijk et al., 2013; Uhl et al., 2016). After confrontation with a threat [such as the content of the movies in our two studies about the consequences of plastics pollution (Study 1) and climate change (Study 2)], people often try to overcome this negative state by regaining stability through behavior that is derived from personal values or social norms (Gray and McNaughton, 2000; McNaughton and Corr, 2004; Harmon-Jones et al., 2009; McGregor et al., 2010; Fritsche et al., 2013; Jonas et al., 2014). Therefore, when people have strong environmental values and identities, they may respond to a freedom threat caused by environmental policies with higher support because it aligns with their values (Fritsche et al., 2010; Bolderdijk et al., 2013). It seems that for environmentally friendly people, expressing support of the initiative facilitates regaining stability.

#### Limitations and Future Research

First, we assessed participants’ resistance to the initiatives with a questionnaire. Future research should address to what extent perspective taking can help influence actual behavior. Second, although imagine-self perspective taking seems to reduce resistance to mandatory actions, the underlying process is unclear. Perspective taking may induce dissonance and personal distress that people subsequently try to reduce by supporting the initiative. Future research should address mediating factors to gain more insights into why imagine-self perspective taking reduces resistance. Third, the initiative we portrayed in our studies included actions that were only distally related to the problems of the person who was affected and whose perspective need to be taken. We speculate that more specific solutions for specific environmental problems would be even more suitable for perspective-taking interventions than the broader, more general sets of solutions that we used in these studies. For example, taking the perspective of children suffering from air pollution should improve the acceptance of a speed limit reduction, but not the acceptance of a disposable plastics ban. Following Berenguer (2007) and Schultz (2000), to implement a ban on disposal cutlery and plastic bags, for instance, participants could be asked to imagine being in the position of a seal suffocating from plastics in the ocean. However, it is also plausible that people generalize across different proenvironmental behaviors, and therefore imperfect matches between problems and solutions may also work to some extent. Furthermore, men were underrepresented in our study samples, thus limiting the generalizability of our findings across genders. Gender differences are plausible because females are better at perspective taking (e.g., Van der Graaff et al., 2014) and tend to exhibit less reactance than males (Seemann et al., 2004). Due to a lack of statistical power to detect a three-way interaction between the manipulated variables and gender, we decided not to test it in the present studies. Another limitation is that our samples consisted of students only. Future research should thus investigate whether the effects can be generalized to the wider public. Finally, the sample was relatively small in general, which is known to generate findings that are hard to replicate. However, the fact that we were able to conceptually replicate the effect in a second study speaks to its robustness. Perspective taking represents only one of many possible interventions to reduce or prevent reactance. Other methods include a restoration postscript after a restriction, telling the participants that they are free to decide if they would like to show a specific behavior (Miller et al., 2007; Bessarabova et al., 2013). Another strategy is inoculation, a note that forewarns of a potential impending freedom threat (Richards et al., 2015). In the future these techniques need to be addressed in the context of environmental policies by systematically testing which is most successful in fostering public support for environmental regulations.

#### Practical Implications

Confronting individuals with threatening climate change information might backfire and result in undesired side effects, such as a lower willingness to show proenvironmental behavior and a higher level of ethnocentrism or outgroup derogation (Fritsche et al., 2012; Uhl et al., 2016, 2017). To optimize political communication and policy making, concepts from behavioral science such as nudging deserve more consideration. Nudging strategies try to alter people’s behavior without forbidding an option. For example, to raise the number of organ donors, the form on which people indicate whether they are willing to function as potential donors was redesigned. As people tend to rely on default options, “yes” was presented as the default option, resulting in a higher number of potential organ donors (Thaler and Sunstein, 2008). Our results show that people can also be nudged when it comes to a mandatory action. Although eliciting reactance at first (significant effect in Study 2), it does not always have to backfire. Imagine-self perspective taking resulted in an even higher support for mandatory actions than voluntary actions. These are the desired and needed results for environmental regulations. Also, real-life examples prove that early resistance can turn into support. For example, when in 2004 smoking was banned from most Irish workplaces including bars and restaurants, public opinion shifted from a previous 59% agreement to 97% agreement after the ban (Action on Smoking and Health, 2004; Office of Tobacco Control, 2004). For environmental policies to be successful we recommend the use of imagine-self perspective taking when implementing a new policy. It could be used in campaigns or politicians could implement it in their speeches (e.g., “Imagine how you would feel…”).

## Conclusion

The aim of this study was to shed further light on individuals’ responses to environmental restrictions. We provide the first evidence that imagine-self perspective taking after a mandatory environmental restriction leads to higher support for the restriction. Furthermore, we found that for high environmentally friendly individuals, an environmental freedom threat might function as an additional motivator to accept environmental actions. Such participants expressed higher support for the actions when they were mandatory than when they were voluntary. We hope that our studies will stimulate future research on the successful implementation of environmental regulations and will help the public finally accept the “urgency of the now.”

## Data Availability Statement

The datasets generated for this study are available on request to the corresponding author.

## Ethics Statement

The studies involving human participants were reviewed and approved by Ethics committee of the University of Salzburg. The patients/participants provided their written informed consent to participate in this study.

## Author Contributions

IU-H, CM, and EJ contributed to conception and design of the study. IU-H and CM organized the data collection. JK performed the statistical analysis. All authors discussed and interpreted the results. IU-H wrote the first draft of the manuscript. IU-H, JK, CM, and EJ wrote sections of the manuscript. All authors contributed to manuscript revision, read and approved the submitted version.

### Conflict of Interest

The authors declare that the research was conducted in the absence of any commercial or financial relationships that could be construed as a potential conflict of interest.
